# Supplementary material for: Elevational pattern of bird species richness and its causes along a central Himalaya gradient, China
Source: PeerJ. 2016 Nov 2;4:e2636. doi: 10.7717/peerj.2636 (PMC5101612; doi:10.7717/peerj.2636)
Supplement: Table S1 — ‘1, 2, 3, … 12’ were the twelve elevational bands distributed from the lowest elevation to the highest elevation along the gradient. [file peerj-04-2636-s001.docx]

**Species checklists of all birds recorded over survey period in the Gyirong Valley.**

| Order | Family | Scientific name | Breeding | Endemic |
| --- | --- | --- | --- | --- |
| Anseriformes | Anatidae | *Anas penelope* |  |  |
| Anseriformes | Anatidae | *Aythya fuligula* |  |  |
| Falconiformes | Accipitridae | *Accipiter badius* | B |  |
| Falconiformes | Accipitridae | *Accipiter trivirgatus* | B |  |
| Falconiformes | Accipitridae | *Aquila chrysaetos* | B |  |
| Falconiformes | Accipitridae | *Aqula clanga* |  |  |
| Falconiformes | Accipitridae | *Aquila nipalensis* | B |  |
| Falconiformes | Accipitridae | *Buteo buteo* | B |  |
| Falconiformes | Accipitridae | *Buteo hemilasius* | B |  |
| Falconiformes | Accipitridae | *Circus cyaneus* |  |  |
| Falconiformes | Accipitridae | *Gypaetus barbatus* | B |  |
| Falconiformes | Accipitridae | *Gyps fulvus* | B |  |
| Falconiformes | Accipitridae | *Gyps himalayensis* | B |  |
| Falconiformes | Accipitridae | *Milvus migrans* | B |  |
| Falconiformes | Accipitridae | *Pernis ptilorhyncus* |  |  |
| Falconiformes | Falconidae | *Falco subbuteo* | B |  |
| Falconiformes | Falconidae | *Falco tinnunculus* | B |  |
| Galliformes | Phasianidae | *Alectoris chukar* | B |  |
| Galliformes | Phasianidae | *Ithaginis cruentus* | B | E |
| Galliformes | Phasianidae | *Lophophorus impejanus* | B | E |
| Galliformes | Phasianidae | *Lophura leucomelanos* | B |  |
| Galliformes | Phasianidae | *Perdix hodgsoniae* | B | E |
| Galliformes | Phasianidae | *Tetraogallus tibetanus* | B |  |
| Gruiformes | Rallidae | *Gallinula chloropus* | B |  |
| Charadriiformes | Ibidorhynchidae | *Ibidorhyncha struthersii* | B |  |
| Charadriiformes | Rostratulidae | *Rostratula benghalensis* | B |  |
| Charadriiformes | Scolopacidae | *Actitis hypoleucos* |  |  |
| Columbiformes | Columbidae | *Columba hodgsonii* | B | E |
| Columbiformes | Columbidae | *Columba leuconota* | B | E |
| Columbiformes | Columbidae | *Columba livia* | B |  |
| Columbiformes | Columbidae | *Columba rupestris* | B |  |
| Columbiformes | Columbidae | *Streptopelia orientalis* | B |  |
| Columbiformes | Columbidae | *Streptopelia turtur* | B |  |
| Columbiformes | Columbidae | *Treron sphenurus* | B |  |
| Cuculiformes | cuculidae | *Cuculus micropterus* | B |  |
| Cuculiformes | cuculidae | *Cuculus sparverioides* | B |  |
| Strigiformes | Strigidae | *Athene noctua* | B |  |
| Strigiformes | Strigidae | *Glaucidium brodiei* | B |  |
| Apodiformes | Apodidae | *Apus nipalensis* | B |  |
| Upupiformes | Upupidae | *Upupa epops* | B |  |
| Piciformes | Capitonidae | *Megalaima virens* | B |  |
| Piciformes | Picidae | *Dendrocopos auriceps* | B |  |
| Piciformes | Picidae | *Dendrocopos cathpharius* | B | E |
| Piciformes | Picidae | *Dendrocopos darjellensis* | B | E |
| Piciformes | Picidae | *Jynx torquilla* | B |  |
| Piciformes | Picidae | *Picus squamatus* | B |  |
| Passeriformes | Alaudidae | *Alauda gulgula* | B |  |
| Passeriformes | Alaudidae | *Calandrella cheleensis* | B |  |
| Passeriformes | Alaudidae | *Eremophila alpestris* | B |  |
| Passeriformes | Hirundinidae | *Hirundo rustica* | B |  |
| Passeriformes | Hirundinidae | *Ptyonoprogne rupestris* | B |  |
| Passeriformes | Hirundinidae | *Riparia riparia* | B |  |
| Passeriformes | Motacillidae | *Anthus hodgsoni* |  |  |
| Passeriformes | Motacillidae | *Anthus roseatus* | B | E |
| Passeriformes | Motacillidae | *Motacilla alba* | B |  |
| Passeriformes | Motacillidae | *Motacilla cinerea* |  |  |
| Passeriformes | Motacillidae | *Motacilla citreola* | B |  |
| Passeriformes | Motacillidae | *Motacilla flava* |  |  |
| Passeriformes | Campephagidae | *Pericrocotus brevirostris* | B | E |
| Passeriformes | Campephagidae | *Pericrocotus ethologus* | B | E |
| Passeriformes | Pycnonotidae | *Hypsipetes leucocephalus* | B |  |
| Passeriformes | Pycnonotidae | *Pycnonotus jocosus* | B |  |
| Passeriformes | Pycnonotidae | *Pycnonotus leucogenys* | B |  |
| Passeriformes | Laniidae | *Lanius tephronotus* | B | E |
| Passeriformes | Oriolidae | *Oriolus traillii* | B |  |
| Passeriformes | Dicruridae | *Dicrurus macrocercus* | B |  |
| Passeriformes | Corvidae | *Cissa chinensis* | B |  |
| Passeriformes | Corvidae | *Corvus corax* | B |  |
| Passeriformes | Corvidae | *Corvus macrorhynchos* | B |  |
| Passeriformes | Corvidae | *Nucifraga caryocatactes* | B |  |
| Passeriformes | Corvidae | *Pyrrhocorax graculus* | B |  |
| Passeriformes | Corvidae | *Pyrrhocorax pyrrhocorax* | B |  |
| Passeriformes | Corvidae | *Urocissa flavirostris* | B | E |
| Passeriformes | Cinclidae | *Cinclus cinclus* | B |  |
| Passeriformes | Cinclidae | *Cinclus pallasii* | B |  |
| Passeriformes | Troglodytidae | *Troglodytes troglodytes* | B |  |
| Passeriformes | Prunellidae | *Prunella fulvescens* | B |  |
| Passeriformes | Prunellidae | *Prunella rubeculoides* | B |  |
| Passeriformes | Prunellidae | *Prunella strophiata* | B | E |
| Passeriformes | Turdidae | *Chaimarrornis leucocephalus* | B | E |
| Passeriformes | Turdidae | *Enicurus scouleri* | B |  |
| Passeriformes | Turdidae | *Grandala coelicolor* | B | E |
| Passeriformes | Turdidae | *Hodgsonius phoenicuroides* | B | E |
| Passeriformes | Turdidae | *Luscinia brunnea* | B | E |
| Passeriformes | Turdidae | *Luscinia pectoralis* | B | E |
| Passeriformes | Turdidae | *Monticola rufiventris* | B |  |
| Passeriformes | Turdidae | *Monticola solitarius* | B |  |
| Passeriformes | Turdidae | *Myophonus caeruleus* | B |  |
| Passeriformes | Turdidae | *Oenanthe deserti* | B |  |
| Passeriformes | Turdidae | *Phoenicurus erythrogaster* | B |  |
| Passeriformes | Turdidae | *Phoenicurus frontalis* | B | E |
| Passeriformes | Turdidae | *Phoenicurus ochruros* | B |  |
| Passeriformes | Turdidae | *Rhyacornis fuliginosus* | B |  |
| Passeriformes | Turdidae | *Saxicola ferreus* | B |  |
| Passeriformes | Turdidae | *Saxicola torquata* | B |  |
| Passeriformes | Turdidae | *Tarsiger chrysaeus* | B | E |
| Passeriformes | Turdidae | *Tarsiger cyanurus* | B |  |
| Passeriformes | Turdidae | *Tarsiger indicus* | B | E |
| Passeriformes | Turdidae | *Turdus albocinctus* | B | E |
| Passeriformes | Turdidae | *Turdus merula* | B |  |
| Passeriformes | Turdidae | *Turdus ruficollis* |  |  |
| Passeriformes | Muscicapidae | *Culicicapa ceylonensis* | B |  |
| Passeriformes | Muscicapidae | *Eumyias thalassina* | B |  |
| Passeriformes | Muscicapidae | *Ficedula hyperythra* | B | E |
| Passeriformes | Muscicapidae | *Ficedula strophiata* | B |  |
| Passeriformes | Muscicapidae | *Ficedula superciliaris* | B |  |
| Passeriformes | Muscicapidae | *Ficedula tricolor* | B | E |
| Passeriformes | Muscicapidae | *Muscicapa dauurica* | B |  |
| Passeriformes | Muscicapidae | *Muscicapa sibirica* | B |  |
| Passeriformes | Muscicapidae | *Niltava sundara* | B | E |
| Passeriformes | Rhipiduridae | *Rhipidura hypoxantha* | B | E |
| Passeriformes | Timaliidae | *Alcippe vinipectus* | B | E |
| Passeriformes | Timaliidae | *Garrulax affinis* | B | E |
| Passeriformes | Timaliidae | *Garrulax erythrocephalus* | B | E |
| Passeriformes | Timaliidae | *Garrulax lineatus* | B | E |
| Passeriformes | Timaliidae | *Garrulax ocellatus* | B | E |
| Passeriformes | Timaliidae | *Garrulax striatus* | B | E |
| Passeriformes | Timaliidae | *Garrulax variegatus* | B | E |
| Passeriformes | Timaliidae | *Heterophasia capistrata* | B | E |
| Passeriformes | Timaliidae | *Minla strigula* | B | E |
| Passeriformes | Timaliidae | *Pnoepyga albiventer* | B | E |
| Passeriformes | Timaliidae | *Pteruthius xanthochlorus* | B | E |
| Passeriformes | Timaliidae | *Yuhina gularis* | B | E |
| Passeriformes | Timaliidae | *Yuhina flavicollis* | B | E |
| Passeriformes | Timaliidae | *Yuhina occipitalis* | B | E |
| Passeriformes | Cisticolidae | *Prinia criniger* | B |  |
| Passeriformes | Cisticolidae | *Prinia hodgsonii* | B |  |
| Passeriformes | Sylviidae | *Cettia brunnifrons* | B | E |
| Passeriformes | Sylviidae | *Cettia flavolivaceus* | B | E |
| Passeriformes | Sylviidae | *Cettia major* | B | E |
| Passeriformes | Sylviidae | *Cettia pallidipes* | B |  |
| Passeriformes | Sylviidae | *Phylloscopus affinis* | B | E |
| Passeriformes | Sylviidae | *Phylloscopus borealis* |  |  |
| Passeriformes | Sylviidae | *Phylloscopus chloronotus* |  |  |
| Passeriformes | Sylviidae | *Phylloscopus fuscatus* | B |  |
| Passeriformes | Sylviidae | *Phylloscopus humei* | B |  |
| Passeriformes | Sylviidae | *Phylloscopus inornatus* |  |  |
| Passeriformes | Sylviidae | *Phylloscopus maculipennis* | B | E |
| Passeriformes | Sylviidae | *Phylloscopus magnirostris* | B | E |
| Passeriformes | Sylviidae | *Phylloscopus pulcher* | B | E |
| Passeriformes | Sylviidae | *Phylloscopus reguloides* | B |  |
| Passeriformes | Sylviidae | *Phylloscopus trochiloides* | B |  |
| Passeriformes | Sylviidae | *Seicercus burkii* | B |  |
| Passeriformes | Sylviidae | *Seicercus xanthoschistos* | B | E |
| Passeriformes | Sylviidae | *Tesia castaneocoronata* | B | E |
| Passeriformes | Zosteropidae | *Zosterops japonicus* | B |  |
| Passeriformes | Aegithalidae | *Aegithalos concinnus* | B |  |
| Passeriformes | Aegithalidae | *Aegithalos iouschistos* | B | E |
| Passeriformes | Paridae | *Parus ater* | B |  |
| Passeriformes | Paridae | *Parus dichrous* | B | E |
| Passeriformes | Paridae | *Parus major* | B |  |
| Passeriformes | Paridae | *Parus monticolus* | B |  |
| Passeriformes | Paridae | *Parus rubidiventris* | B | E |
| Passeriformes | Paridae | *Pseudopodoces humilis* | B |  |
| Passeriformes | Sittidae | *Sitta himalayensis* | B | E |
| Passeriformes | Trichodoninae | *Tichodroma muraria* | B |  |
| Passeriformes | Certhiidae | *Certhia familiaris* | B |  |
| Passeriformes | Certhiidae | *Certhia nipalensis* | B | E |
| Passeriformes | Dicaeidae | *Dicaeum ignipectus* | B |  |
| Passeriformes | Bombycillidae | *Aethopyga gouldiae* | B |  |
| Passeriformes | Bombycillidae | *Aethopyga ignicauda* | B | E |
| Passeriformes | Bombycillidae | *Aethopyga nipalensis* | B | E |
| Passeriformes | Passeridae | *Montifringilla adamsi* | B |  |
| Passeriformes | Passeridae | *Passer montanus* | B |  |
| Passeriformes | Frigillidae | *Carpodacus edwardsii* | B | E |
| Passeriformes | Frigillidae | *Carpodacus erythrinus* | B |  |
| Passeriformes | Frigillidae | *Carpodacus nipalensis* | B | E |
| Passeriformes | Frigillidae | *Carpodacus pulcherrimus* | B | E |
| Passeriformes | Frigillidae | *Carpodacus puniceus* | B |  |
| Passeriformes | Frigillidae | *Carpodacus rhodopeplus* | B | E |
| Passeriformes | Frigillidae | *Carpodacus rodochroa* | B | E |
| Passeriformes | Frigillidae | *Carpodacus rubicilla* | B |  |
| Passeriformes | Frigillidae | *Carpodacus rubicilloides* | B |  |
| Passeriformes | Frigillidae | *Carpodacus thura* | B | E |
| Passeriformes | Frigillidae | *Carduelis flavirostris* | B |  |
| Passeriformes | Frigillidae | *Carduelis spinoides* | B |  |
| Passeriformes | Frigillidae | *Haematospiza sipahi* | B | E |
| Passeriformes | Frigillidae | *Leucosticte brandti* | B |  |
| Passeriformes | Frigillidae | *Mycerobas carnipes* | B |  |
| Passeriformes | Frigillidae | *Pinicola subhimachala* | B | E |
| Passeriformes | Frigillidae | *Pyrrhula erythrocephala* | B | E |
| Passeriformes | Frigillidae | *Serinus pusillus* | B |  |

Endemic species were defined based on their distributions limited to the Himalayas and adjacent Hengduan mountains.
