# Supplementary material for: Elevational pattern of bird species richness and its causes along a central Himalaya gradient, China
Source: PeerJ. 2016 Nov 2;4:e2636. doi: 10.7717/peerj.2636 (PMC5101612; doi:10.7717/peerj.2636)
Supplement: Table S2 [file peerj-04-2636-s002.docx]

**Observed, estimated (Chao2, Jackknife2) and interpolated richness of birds in each elevational band in the Giyrong Valley.**

| **Elevation bands (m)** | **Observed richness** | **Chao2 ± SD** | **Jackknife2** | **Interpolated richness** |
| --- | --- | --- | --- | --- |
| **1800-2100** | 45 | 56.25 ± 8.68 | 64 | 45 |
| **2100-2400** | 44 | 49.25 ± 3.85 | 58.08 | 53 |
| **2400-2700** | 58 | 76.75 ± 0.21 | 85.58 | 74 |
| **2700-3000** | 61 | 88.68 ± 4.16 | 93.92 | 79 |
| **3000-3300** | 56 | 70.44 ± 8.37 | 79.83 | 73 |
| **3300-3600** | 62 | 77.23 ± 8.12 | 89.5 | 74 |
| **3600-3900** | 61 | 90.06 ± 4.36 | 96.08 | 71 |
| **3900-4200** | 48 | 82.17 ± 8.79 | 79.08 | 57 |
| **4200-4500** | 35 | 41.19 ± 4.93 | 47.67 | 41 |
| **4500-4800** | 30 | 38.25 ± 6.81 | 42.42 | 33 |
| **4800-5100** | 25 | 28.86 ± 3.63 | 34.25 | 26 |
| **5100-5400** | 13 | 13.32 ± 0.74 | 14.75 | 13 |
| **Overall** | 169 | 210.25 ± 5.1 | 229.08 | 169 |
