# Supplementary material for: Elevational pattern of bird species richness and its causes along a central Himalaya gradient, China
Source: PeerJ. 2016 Nov 2;4:e2636. doi: 10.7717/peerj.2636 (PMC5101612; doi:10.7717/peerj.2636)
Supplement: Table S3 [file peerj-04-2636-s003.docx]

**Polynomial regressions of the interpolated species richness patterns along the elevational gradients for all the species groups.**

| **Regressions** | **Overall species** | **Large-ranged species** | **Small-ranged species** |
| --- | --- | --- | --- |
| **First-order *r*^2^** | 0.421 | 0.497* | 0.309 |
| **AICc** | 3033.5* | 722.9 | 928.07 |
| **Second-order *r*^2^** | 0.922** | 0.873** | 0.896** |
| **AICc** | 412.62 | 187.34 | 144.42 |
| **Third-order *r*^2^** | **0.972**** | **0.949**** | **0.922**** |
| **AICc** | **152.79** | **81.53** | **112.81** |

AIC_c_, corrected Akaike information criterion. **P* < 0.05, ***P* < 0.01; Bold numbers indicate the best regression model with lowest AICc value.
