# Supplementary material for: Elevational pattern of bird species richness and its causes along a central Himalaya gradient, China
Source: PeerJ. 2016 Nov 2;4:e2636. doi: 10.7717/peerj.2636 (PMC5101612; doi:10.7717/peerj.2636)
Supplement: Table S4 [file peerj-04-2636-s004.docx]

**Parameter estimates averaged across 63 ordinary least squares (OLS) models.**

|  | **Standard coefficient of the model averaging** | | | | | | | |
| --- | --- | --- | --- | --- | --- | --- | --- | --- |
| **Species groups** | **Area** | **MAT** | **MAP** | **NDVI** | **HH** | **MDE** | ***r*^2^_adj_** | **AICc** |
| **Overall birds** | -0.48 | -0.218 | -0.124 | 0.781 | 0.569 | 0.024 | 0.893 | 138.718 |
| **Large-ranged species** | -0.322 | -0.583 | -0.224 | 1.351 | 0.246 | 0.325 | 0.781 | 131.755 |
| **Small-ranged species** | -0.489 | 0.519 | 0.435 | 0.529 | 0.801 | -0.229 | -1.846 | 161.741 |

MAT, mean annual temperature; MAP, mean annual precipitation; NDVI, normalized difference vegetation index; HH, habitat heterogeneity; MDE, the mid-domain effect; AIC_c_, corrected Akaike information criterion. *r*^2^_adj_ is the adjusted *r*^2^ value for multiple regressions. Negative relationships are indicated by ‘-’..
