# Supplementary material for: Elevational pattern of bird species richness and its causes along a central Himalaya gradient, China
Source: PeerJ. 2016 Nov 2;4:e2636. doi: 10.7717/peerj.2636 (PMC5101612; doi:10.7717/peerj.2636)
Supplement: Table S5 [file peerj-04-2636-s005.docx]

**All 63 regression models for interpolated species richness of different species groups of birds (overall species, lagre-ranged species, small-ranged species).**

**Table S5-1** Sixty-three regression models for interpolated species richness of all breeding birds in the Gyirong Valley. Explanatory variables are: 1, area; 2, mean annual temperature; 3,mean annual precipitation; 4, normalized difference vegetation index; 5, habitat heterogeneity; and 6, mid-domain effect. Regression results are sorted by corrected Akaike information criterion (AIC_c_). nVars., the number of parameters in each model; Cond.Num., the condition number in each model, a measure of multicollinearity, and a lower value indicates less multicollinearity; *L*(*gi*|*x*), the model likelihood; AIC_c_ wi., corrected AIC weightings.

| Model | Variables | nVars. | *r*² | Cond. Num. | AIC_c_ | Delta AIC_c_ | L(*gi\|x*) | AIC_c_ wi |
| --- | --- | --- | --- | --- | --- | --- | --- | --- |
| 60 | 4, 5 | 2 | 0.989 | 1.149 | 66.702 | 0 | 1 | 0.573 |
| 45 | 2, 4, 5 | 3 | 0.992 | 7.437 | 68.595 | 1.893 | 0.388 | 0.223 |
| 53 | 3, 4, 5 | 3 | 0.991 | 5.938 | 70.863 | 4.161 | 0.125 | 0.072 |
| 32 | 1, 5 | 2 | 0.983 | 1.13 | 71.778 | 5.076 | 0.079 | 0.045 |
| 29 | 1, 4, 5 | 3 | 0.99 | 13.577 | 72.188 | 5.486 | 0.064 | 0.037 |
| 59 | 4, 5, 6 | 3 | 0.989 | 6.886 | 72.962 | 6.26 | 0.044 | 0.025 |
| 14 | 1, 2, 4, 5 | 4 | 0.993 | 16.647 | 75.661 | 8.959 | 0.011 | 0.007 |
| 44 | 2, 4, 5, 6 | 4 | 0.992 | 8.356 | 77.082 | 10.38 | 0.006 | 0.003 |
| 38 | 2, 3, 4, 5 | 4 | 0.992 | 12.479 | 77.37 | 10.667 | 0.005 | 0.003 |
| 25 | 1, 3, 5 | 3 | 0.984 | 5.877 | 77.45 | 10.748 | 0.005 | 0.003 |
| 31 | 1, 5, 5 | 3 | 0.984 | 6.864 | 77.535 | 10.832 | 0.004 | 0.003 |
| 17 | 1, 2, 5 | 3 | 0.984 | 6.774 | 77.699 | 10.997 | 0.004 | 0.002 |
| 22 | 1, 3, 4, 5 | 4 | 0.992 | 15.931 | 77.964 | 11.261 | 0.004 | 0.002 |
| 52 | 3, 4, 5, 6 | 4 | 0.992 | 10.248 | 78.428 | 11.726 | 0.003 | 0.002 |
| 28 | 1, 4, 5, 6 | 4 | 0.99 | 14.68 | 80.86 | 14.158 | <.001 | <.001 |
| 30 | 1, 6 | 2 | 0.963 | 1.13 | 81.281 | 14.579 | <.001 | <.001 |
| 58 | 4, 6 | 2 | 0.962 | 1.159 | 81.37 | 14.668 | <.001 | <.001 |
| 16 | 1, 2, 5, 6 | 4 | 0.984 | 7.233 | 86.097 | 19.395 | <.001 | <.001 |
| 24 | 1, 3, 5, 6 | 4 | 0.984 | 9.174 | 86.126 | 19.424 | <.001 | <.001 |
| 10 | 1, 2, 3, 5 | 4 | 0.984 | 11.766 | 86.25 | 19.548 | <.001 | <.001 |
| 27 | 1, 4, 6 | 3 | 0.965 | 13.691 | 86.661 | 19.959 | <.001 | <.001 |
| 43 | 2, 4, 6 | 3 | 0.965 | 7.833 | 86.898 | 20.196 | <.001 | <.001 |
| 15 | 1, 2, 6 | 3 | 0.963 | 6.898 | 87.431 | 20.729 | <.001 | <.001 |
| 23 | 1, 3, 6 | 3 | 0.963 | 7.029 | 87.508 | 20.805 | <.001 | <.001 |
| 51 | 3, 4, 6 | 3 | 0.962 | 7.554 | 87.656 | 20.954 | <.001 | <.001 |
| 13 | 1, 2, 4, 5, 6 | 5 | 0.993 | 17.525 | 88.736 | 22.034 | <.001 | <.001 |
| 48 | 2, 5 | 2 | 0.93 | 1.085 | 88.764 | 22.062 | <.001 | <.001 |
| 7 | 1, 2, 3, 4, 5 | 5 | 0.993 | 19.677 | 88.856 | 22.154 | <.001 | <.001 |
| 21 | 1, 3, 4, 5, 6 | 5 | 0.993 | 17.014 | 90.079 | 23.377 | <.001 | <.001 |
| 54 | 3, 6 | 2 | 0.922 | 1.445 | 90.122 | 23.42 | <.001 | <.001 |
| 37 | 2, 3, 4, 5, 6 | 5 | 0.993 | 16.027 | 90.189 | 23.487 | <.001 | <.001 |
| 46 | 2, 6 | 2 | 0.912 | 1.092 | 91.559 | 24.857 | <.001 | <.001 |
| 56 | 3, 5 | 2 | 0.906 | 1.389 | 92.303 | 25.601 | <.001 | <.001 |
| 50 | 3, 4 | 2 | 0.892 | 3.358 | 94.041 | 27.339 | <.001 | <.001 |
| 47 | 2, 5, 6 | 3 | 0.934 | 6.839 | 94.297 | 27.594 | <.001 | <.001 |
| 36 | 2, 3, 4, 6 | 4 | 0.968 | 13.884 | 94.489 | 27.787 | <.001 | <.001 |
| 12 | 1, 2, 4, 6 | 4 | 0.968 | 17.06 | 94.552 | 27.85 | <.001 | <.001 |
| 55 | 3, 5, 6 | 3 | 0.933 | 7.206 | 94.607 | 27.905 | <.001 | <.001 |
| 41 | 2, 3, 5 | 3 | 0.93 | 9.448 | 95.046 | 28.344 | <.001 | <.001 |
| 8 | 1, 2, 3, 6 | 4 | 0.966 | 13.799 | 95.357 | 28.655 | <.001 | <.001 |
| 20 | 1, 3, 4, 6 | 4 | 0.965 | 16.282 | 95.448 | 28.745 | <.001 | <.001 |
| 39 | 2, 3, 6 | 3 | 0.922 | 11.7 | 96.324 | 29.622 | <.001 | <.001 |
| 18 | 1, 3 | 2 | 0.866 | 3.429 | 96.596 | 29.894 | <.001 | <.001 |
| 9 | 1, 2, 3, 5, 6 | 5 | 0.984 | 16.054 | 99.288 | 32.586 | <.001 | <.001 |
| 19 | 1, 3, 4 | 3 | 0.895 | 15.873 | 99.902 | 33.2 | <.001 | <.001 |
| 42 | 2, 4 | 2 | 0.819 | 5.686 | 100.162 | 33.46 | <.001 | <.001 |
| 35 | 2, 3, 4 | 3 | 0.892 | 11.995 | 100.253 | 33.551 | <.001 | <.001 |
| 3 | 1, 2, 3 | 3 | 0.876 | 10.835 | 101.938 | 35.236 | <.001 | <.001 |
| 40 | 2, 3, 5, 6 | 4 | 0.937 | 14.569 | 102.616 | 35.914 | <.001 | <.001 |
| 57 | 4 | 1 | 0.654 | 1 | 103.246 | 36.544 | <.001 | <.001 |
| 2 | 1, 2 | 2 | 0.755 | 5.516 | 103.849 | 37.147 | <.001 | <.001 |
| 1 | 1 | 1 | 0.632 | 1 | 103.991 | 37.288 | <.001 | <.001 |
| 34 | 2, 3 | 2 | 0.699 | 6.098 | 106.306 | 39.604 | <.001 | <.001 |
| 11 | 1, 2, 4 | 3 | 0.82 | 16.236 | 106.415 | 39.712 | <.001 | <.001 |
| 5 | 1, 2, 3, 4, 6 | 5 | 0.97 | 19.657 | 106.724 | 40.021 | <.001 | <.001 |
| 26 | 1, 4 | 2 | 0.655 | 13.381 | 107.924 | 41.222 | <.001 | <.001 |
| 63 | 5 | 1 | 0.468 | 1 | 108.41 | 41.708 | <.001 | <.001 |
| 4 | 1, 2, 3, 4 | 4 | 0.896 | 19.648 | 108.601 | 41.899 | <.001 | <.001 |
| 61 | 6 | 1 | 0.446 | 1 | 108.912 | 42.21 | <.001 | <.001 |
| 33 | 2 | 1 | 0.386 | 1 | 110.129 | 43.427 | <.001 | <.001 |
| 6 | 1, 2, 3, 4, 5, 6 | 6 | 0.993 | 19.826 | 110.561 | 43.859 | <.001 | <.001 |
| 62 | 5, 6 | 2 | 0.47 | 6.812 | 113.084 | 46.382 | <.001 | <.001 |
| 49 | 3 | 1 | 0.169 | 1 | 113.776 | 47.074 | <.001 | <.001 |

**Table S5-2** Sixty-three regression models for interpolated species richness of large-ranged birds in the Gyirong Valley. Explanatory variables are: 1, area; 2, mean annual temperature; 3,mean annual precipitation; 4, normalized difference vegetation index; 5, habitat heterogeneity; and 6, mid-domain effect. Regression results are sorted by corrected Akaike information criterion (AIC_c_). nVars., the number of parameters in each model; Cond.Num., the condition number in each model, a measure of multicollinearity, and a lower value indicates less multicollinearity; *L*(*gi*|*x*), the model likelihood; AIC_c_ wi., corrected AIC weightings.

| Model | Variables | nVars. | *r*² | Cond. Num. | | AIC_c_ | Delta AIC_c_ | L(*gi\|x*) | AIC_c_ wi |
| --- | --- | --- | --- | --- | --- | --- | --- | --- | --- |
| 45 | 2, 4, 6 | 3 | 0.993 | 8.213 | 52.405 | | 0 | 1 | 0.74 |
| 43 | 2, 4, 5 | 3 | 0.99 | 7.437 | 56.034 | | 3.628 | 0.163 | 0.121 |
| 14 | 1, 2, 4, 6 | 4 | 0.994 | 16.922 | 57.859 | | 5.454 | 0.065 | 0.048 |
| 44 | 2, 4, 5, 6 | 4 | 0.994 | 8.73 | 58.16 | | 5.755 | 0.056 | 0.042 |
| 38 | 2, 3, 4, 6 | 4 | 0.993 | 14.133 | 60.568 | | 8.162 | 0.017 | 0.013 |
| 60 | 4, 6 | 2 | 0.974 | 1.165 | 61.29 | | 8.884 | 0.012 | 0.009 |
| 51 | 3, 4, 5 | 3 | 0.984 | 5.938 | 61.676 | | 9.27 | 0.01 | 0.007 |
| 36 | 2, 3, 4, 5 | 4 | 0.992 | 12.479 | 62.567 | | 10.161 | 0.006 | 0.005 |
| 12 | 1, 2, 4, 5 | 4 | 0.992 | 16.647 | 62.815 | | 10.41 | 0.005 | 0.004 |
| 53 | 3, 4, 6 | 3 | 0.982 | 8.449 | 63.314 | | 10.908 | 0.004 | 0.003 |
| 50 | 3, 4 | 2 | 0.968 | 3.358 | 63.842 | | 11.436 | 0.003 | 0.002 |
| 32 | 1, 6 | 2 | 0.968 | 1.142 | 63.923 | | 11.518 | 0.003 | 0.002 |
| 13 | 1, 2, 4, 5, 6 | 5 | 0.996 | 17.046 | 67.051 | | 14.645 | <.001 | <.001 |
| 29 | 1, 4, 6 | 3 | 0.975 | 13.63 | 67.078 | | 14.672 | <.001 | <.001 |
| 17 | 1, 2, 6 | 3 | 0.975 | 7.244 | 67.311 | | 14.906 | <.001 | <.001 |
| 59 | 4, 5, 6 | 3 | 0.974 | 5.706 | 67.378 | | 14.972 | <.001 | <.001 |
| 58 | 4, 5 | 2 | 0.955 | 1.149 | 67.938 | | 15.533 | <.001 | <.001 |
| 20 | 1, 3, 4, 5 | 4 | 0.987 | 15.931 | 68.045 | | 15.639 | <.001 | <.001 |
| 19 | 1, 3, 4 | 3 | 0.972 | 15.873 | 68.463 | | 16.057 | <.001 | <.001 |
| 35 | 2, 3, 4 | 3 | 0.971 | 11.995 | 68.737 | | 16.332 | <.001 | <.001 |
| 25 | 1, 3, 6 | 3 | 0.971 | 7.999 | 68.871 | | 16.465 | <.001 | <.001 |
| 23 | 1, 3, 5 | 3 | 0.969 | 5.877 | 69.789 | | 17.384 | <.001 | <.001 |
| 31 | 1, 5, 6 | 3 | 0.968 | 5.691 | 70.103 | | 17.697 | <.001 | <.001 |
| 30 | 1, 5 | 2 | 0.946 | 1.13 | 70.156 | | 17.751 | <.001 | <.001 |
| 52 | 3, 4, 5, 6 | 4 | 0.985 | 10.711 | 70.16 | | 17.754 | <.001 | <.001 |
| 22 | 1, 3, 4, 6 | 4 | 0.984 | 16.085 | 70.313 | | 17.908 | <.001 | <.001 |
| 18 | 1, 3 | 2 | 0.943 | 3.429 | 70.743 | | 18.337 | <.001 | <.001 |
| 7 | 1, 2, 3, 4, 6 | 5 | 0.995 | 19.765 | 70.8 | | 18.394 | <.001 | <.001 |
| 37 | 2, 3, 4, 5, 6 | 5 | 0.994 | 15.526 | 71.275 | | 18.869 | <.001 | <.001 |
| 42 | 2, 4 | 2 | 0.939 | 5.686 | 71.47 | | 19.064 | <.001 | <.001 |
| 15 | 1, 2, 5 | 3 | 0.964 | 6.774 | 71.661 | | 19.255 | <.001 | <.001 |
| 5 | 1, 2, 3, 4, 5 | 5 | 0.994 | 19.677 | 71.969 | | 19.564 | <.001 | <.001 |
| 27 | 1, 4, 5 | 3 | 0.955 | 13.577 | 74.095 | | 21.689 | <.001 | <.001 |
| 28 | 1, 4, 5, 6 | 4 | 0.976 | 14.325 | 75.691 | | 23.285 | <.001 | <.001 |
| 16 | 1, 2, 5, 6 | 4 | 0.975 | 7.656 | 75.781 | | 23.376 | <.001 | <.001 |
| 4 | 1, 2, 3, 4 | 4 | 0.975 | 19.648 | 75.86 | | 23.455 | <.001 | <.001 |
| 10 | 1, 2, 3, 6 | 4 | 0.975 | 14.274 | 76.056 | | 23.651 | <.001 | <.001 |
| 3 | 1, 2, 3 | 3 | 0.944 | 10.835 | 76.912 | | 24.506 | <.001 | <.001 |
| 24 | 1, 3, 5, 6 | 4 | 0.972 | 10.019 | 77.132 | | 24.727 | <.001 | <.001 |
| 56 | 3, 6 | 2 | 0.9 | 1.46 | 77.446 | | 25.04 | <.001 | <.001 |
| 11 | 1, 2, 4 | 3 | 0.941 | 16.236 | 77.526 | | 25.121 | <.001 | <.001 |
| 8 | 1, 2, 3, 5 | 4 | 0.969 | 11.766 | 78.353 | | 25.947 | <.001 | <.001 |
| 48 | 2, 6 | 2 | 0.887 | 1.099 | 79.008 | | 26.602 | <.001 | <.001 |
| 2 | 1, 2 | 2 | 0.873 | 5.516 | 80.352 | | 27.947 | <.001 | <.001 |
| 21 | 1, 3, 4, 5, 6 | 5 | 0.987 | 16.204 | 81.03 | | 28.625 | <.001 | <.001 |
| 57 | 4 | 1 | 0.763 | 1 | 83.115 | | 30.709 | <.001 | <.001 |
| 55 | 3, 5, 6 | 3 | 0.902 | 5.985 | 83.517 | | 31.111 | <.001 | <.001 |
| 46 | 2, 5 | 2 | 0.833 | 1.085 | 83.633 | | 31.228 | <.001 | <.001 |
| 41 | 2, 3, 6 | 3 | 0.901 | 12.012 | 83.707 | | 31.301 | <.001 | <.001 |
| 1 | 1 | 1 | 0.741 | 1 | 84.214 | | 31.808 | <.001 | <.001 |
| 47 | 2, 5, 6 | 3 | 0.887 | 5.668 | 85.289 | | 32.884 | <.001 | <.001 |
| 54 | 3, 5 | 2 | 0.79 | 1.389 | 86.375 | | 33.969 | <.001 | <.001 |
| 26 | 1, 4 | 2 | 0.764 | 13.381 | 87.803 | | 35.397 | <.001 | <.001 |
| 9 | 1, 2, 3, 5, 6 | 5 | 0.975 | 15.844 | 88.979 | | 36.574 | <.001 | <.001 |
| 6 | 1, 2, 3, 4, 5, 6 | 6 | 0.996 | 19.872 | 89.031 | | 36.625 | <.001 | <.001 |
| 39 | 2, 3, 5 | 3 | 0.836 | 9.448 | 89.7 | | 37.295 | <.001 | <.001 |
| 34 | 2, 3 | 2 | 0.707 | 6.098 | 90.409 | | 38.004 | <.001 | <.001 |
| 40 | 2, 3, 5, 6 | 4 | 0.902 | 13.9 | 92.315 | | 39.91 | <.001 | <.001 |
| 33 | 2 | 1 | 0.46 | 1 | 93.017 | | 40.612 | <.001 | <.001 |
| 63 | 6 | 1 | 0.344 | 1 | 95.353 | | 42.947 | <.001 | <.001 |
| 61 | 5 | 1 | 0.307 | 1 | 96.009 | | 43.604 | <.001 | <.001 |
| 49 | 3 | 1 | 0.234 | 1 | 97.205 | | 44.8 | <.001 | <.001 |
| 62 | 5, 6 | 2 | 0.344 | 5.64 | 100.065 | | 47.66 | <.001 | <.001 |

**Table S5-3** Sixty-three regression models for interpolated species richness of small-ranged birds in the Gyirong Valley. Explanatory variables are: 1, area; 2, mean annual temperature; 3,mean annual precipitation; 4, normalized difference vegetation index; 5, habitat heterogeneity; and 6, mid-domain effect. Regression results are sorted by corrected Akaike information criterion (AIC_c_). nVars., the number of parameters in each model; Cond.Num., the condition number in each model, a measure of multicollinearity, and a lower value indicates less multicollinearity; *L*(*gi*|*x*), the model likelihood; AIC_c_ wi., corrected AIC weightings.

| Model | Variables | nVars. | *r*² | Cond. Num. | AIC_c_ | Delta AIC_c_ | L(*gi\|x*) | AIC_c_ wi |
| --- | --- | --- | --- | --- | --- | --- | --- | --- |
| 58 | 4, 5 | 2 | 0.966 | 1.149 | 63.916 | 0 | 1 | 0.246 |
| 46 | 2, 5 | 2 | 0.965 | 1.085 | 64.206 | 0.29 | 0.865 | 0.213 |
| 30 | 1, 5 | 2 | 0.963 | 1.13 | 64.77 | 0.854 | 0.652 | 0.161 |
| 54 | 3, 5 | 2 | 0.96 | 1.389 | 65.78 | 1.864 | 0.394 | 0.097 |
| 51 | 3, 4, 5 | 3 | 0.974 | 5.938 | 66.752 | 2.837 | 0.242 | 0.06 |
| 23 | 1, 3, 5 | 3 | 0.973 | 5.877 | 67.316 | 3.4 | 0.183 | 0.045 |
| 59 | 4, 5, 6 | 3 | 0.972 | 7.856 | 67.527 | 3.611 | 0.164 | 0.04 |
| 15 | 1, 2, 5 | 3 | 0.972 | 6.774 | 67.826 | 3.91 | 0.142 | 0.035 |
| 43 | 2, 4, 5 | 3 | 0.972 | 7.437 | 67.839 | 3.923 | 0.141 | 0.035 |
| 47 | 2, 5, 6 | 3 | 0.968 | 7.824 | 69.211 | 5.295 | 0.071 | 0.017 |
| 39 | 2, 3, 5 | 3 | 0.967 | 9.448 | 69.69 | 5.774 | 0.056 | 0.014 |
| 27 | 1, 4, 5 | 3 | 0.966 | 13.577 | 69.879 | 5.963 | 0.051 | 0.012 |
| 31 | 1, 5, 6 | 3 | 0.965 | 7.868 | 70.273 | 6.357 | 0.042 | 0.01 |
| 55 | 3, 5, 6 | 3 | 0.96 | 8.228 | 72.055 | 8.139 | 0.017 | 0.004 |
| 44 | 2, 4, 5, 6 | 4 | 0.977 | 7.963 | 74.212 | 10.296 | 0.006 | 0.001 |
| 52 | 3, 4, 5, 6 | 4 | 0.976 | 9.023 | 74.478 | 10.562 | 0.005 | 0.001 |
| 32 | 1, 6 | 2 | 0.915 | 1.101 | 74.787 | 10.871 | 0.004 | 0.001 |
| 16 | 1, 2, 5, 6 | 4 | 0.975 | 8.165 | 75.346 | 11.43 | 0.003 | <.001 |
| 20 | 1, 3, 4, 5 | 4 | 0.974 | 15.931 | 75.466 | 11.55 | 0.003 | <.001 |
| 36 | 2, 3, 4, 5 | 4 | 0.974 | 12.479 | 75.498 | 11.582 | 0.003 | <.001 |
| 8 | 1, 2, 3, 5 | 4 | 0.974 | 11.766 | 75.821 | 11.905 | 0.003 | <.001 |
| 60 | 4, 6 | 2 | 0.907 | 1.135 | 75.884 | 11.968 | 0.003 | <.001 |
| 24 | 1, 3, 5, 6 | 4 | 0.973 | 8.396 | 75.911 | 11.995 | 0.002 | <.001 |
| 28 | 1, 4, 5, 6 | 4 | 0.972 | 15.974 | 76.323 | 12.408 | 0.002 | <.001 |
| 12 | 1, 2, 4, 5 | 4 | 0.972 | 16.647 | 76.408 | 12.493 | 0.002 | <.001 |
| 56 | 3, 6 | 2 | 0.902 | 1.44 | 76.483 | 12.568 | 0.002 | <.001 |
| 48 | 2, 6 | 2 | 0.897 | 1.101 | 77.091 | 13.175 | 0.001 | <.001 |
| 40 | 2, 3, 5, 6 | 4 | 0.969 | 12.764 | 77.86 | 13.944 | <.001 | <.001 |
| 25 | 1, 3, 6 | 3 | 0.921 | 6.111 | 80.198 | 16.283 | <.001 | <.001 |
| 17 | 1, 2, 6 | 3 | 0.917 | 6.595 | 80.786 | 16.87 | <.001 | <.001 |
| 29 | 1, 4, 6 | 3 | 0.915 | 13.766 | 81.073 | 17.157 | <.001 | <.001 |
| 53 | 3, 4, 6 | 3 | 0.914 | 6.601 | 81.154 | 17.238 | <.001 | <.001 |
| 45 | 2, 4, 6 | 3 | 0.909 | 7.5 | 81.822 | 17.907 | <.001 | <.001 |
| 41 | 2, 3, 6 | 3 | 0.903 | 10.736 | 82.58 | 18.665 | <.001 | <.001 |
| 37 | 2, 3, 4, 5, 6 | 5 | 0.977 | 14.45 | 87.278 | 23.362 | <.001 | <.001 |
| 13 | 1, 2, 4, 5, 6 | 5 | 0.977 | 18.815 | 87.398 | 23.482 | <.001 | <.001 |
| 21 | 1, 3, 4, 5, 6 | 5 | 0.976 | 18.432 | 87.672 | 23.756 | <.001 | <.001 |
| 61 | 5 | 1 | 0.611 | 1 | 88.285 | 24.369 | <.001 | <.001 |
| 9 | 1, 2, 3, 5, 6 | 5 | 0.975 | 14.662 | 88.391 | 24.475 | <.001 | <.001 |
| 50 | 3, 4 | 2 | 0.733 | 3.358 | 88.489 | 24.574 | <.001 | <.001 |
| 5 | 1, 2, 3, 4, 5 | 5 | 0.974 | 19.677 | 88.599 | 24.684 | <.001 | <.001 |
| 10 | 1, 2, 3, 6 | 4 | 0.922 | 12.994 | 88.845 | 24.93 | <.001 | <.001 |
| 22 | 1, 3, 4, 6 | 4 | 0.922 | 16.506 | 88.855 | 24.94 | <.001 | <.001 |
| 14 | 1, 2, 4, 6 | 4 | 0.917 | 17.248 | 89.506 | 25.59 | <.001 | <.001 |
| 18 | 1, 3 | 2 | 0.709 | 3.429 | 89.51 | 25.595 | <.001 | <.001 |
| 38 | 2, 3, 4, 6 | 4 | 0.915 | 13.259 | 89.853 | 25.938 | <.001 | <.001 |
| 63 | 6 | 1 | 0.534 | 1 | 90.439 | 26.524 | <.001 | <.001 |
| 57 | 4 | 1 | 0.486 | 1 | 91.617 | 27.701 | <.001 | <.001 |
| 1 | 1 | 1 | 0.467 | 1 | 92.053 | 28.138 | <.001 | <.001 |
| 34 | 2, 3 | 2 | 0.631 | 6.098 | 92.365 | 28.449 | <.001 | <.001 |
| 42 | 2, 4 | 2 | 0.624 | 5.686 | 92.6 | 28.685 | <.001 | <.001 |
| 62 | 5, 6 | 2 | 0.621 | 7.779 | 92.69 | 28.774 | <.001 | <.001 |
| 35 | 2, 3, 4 | 3 | 0.745 | 11.995 | 94.195 | 30.279 | <.001 | <.001 |
| 2 | 1, 2 | 2 | 0.567 | 5.516 | 94.274 | 30.359 | <.001 | <.001 |
| 3 | 1, 2, 3 | 3 | 0.74 | 10.835 | 94.461 | 30.546 | <.001 | <.001 |
| 19 | 1, 3, 4 | 3 | 0.736 | 15.873 | 94.639 | 30.723 | <.001 | <.001 |
| 33 | 2 | 1 | 0.28 | 1 | 95.671 | 31.755 | <.001 | <.001 |
| 26 | 1, 4 | 2 | 0.488 | 13.381 | 96.293 | 32.377 | <.001 | <.001 |
| 49 | 3 | 1 | 0.098 | 1 | 98.382 | 34.467 | <.001 | <.001 |
| 11 | 1, 2, 4 | 3 | 0.624 | 16.236 | 98.883 | 34.968 | <.001 | <.001 |
| 7 | 1, 2, 3, 4, 6 | 5 | 0.922 | 19.732 | 101.971 | 38.055 | <.001 | <.001 |
| 4 | 1, 2, 3, 4 | 4 | 0.749 | 19.648 | 102.814 | 38.898 | <.001 | <.001 |
| 6 | 1, 2, 3, 4, 5, 6 | 6 | 0.977 | 21.402 | 109.266 | 45.351 | <.001 | <.001 |
